# "The Hospital" Nursing Mirror

**Published:** 1900-09-29

**Authors:** 


### "The Hospital" Nursing Mirror.

Being the Nursing Section of "The Hospital."

[Contributions for this Section of "The Hospital" should be addressed to the Editor, The Hospital, 28 & 29, Southampton Street, Strand, London, W.C., and should have the word "Nursing" plainly written in left-hand top corner of the envelope.]

### Notes on News from the Nursing World.

### THE ARMY NURSING SERVICE RESERVE.

It is stated that "the War Office has issued revised regulations for the Army Nursing Service Reserve," and the assumption is drawn "that those in force up to the present time have not proved wholly satisfactory." The regulations of the Reserve are a fair subject for criticism, and there are some of them to which exception might reasonably be taken. But it is only due to the War Office to say that, whether good, bad, or indifferent, no material change has been made in them, at any rate since May 13th, 1899, when we published an account of an interview with the secretary of the Army Nursing Reserve. Then, as now, one of the rules was that the candidates for appointment must not be under 25 nor over 35 years of age, though there is no objection to work being continued long after that age. Then, as now, a certificate of three years' training and service in a civil hospital was the qualification required. Then, as now, two recent testimonials of efficiency in medical and surgical nursing were required from medical officers. Then, as now, a recommendation from a person of social position was needed. Then, as now, the salary given was at the rate of £40 per annum, with a gratuity on cessation of employment. The assumption that the regulations have not worked satisfactorily is, therefore, a little odd; and the attack on Miss Wedgwood for not dissociating herself with the committee a little belated.

### THE ALLEGATIONS OF NURSE ROBERTS.

Prominence has been afforded to the evidence of Nurse Roberts before the Hospital Commissioners. She joined the "Sumatra" at Durban on November 4th. Miss Roberts stated that there was lack of necessaries and comforts and an insufficient number of orderlies on board the "Sumatra" and two other transports. She averred that on the way to Cape Town from Natal the men on the "Sumatra" had only bread and tea, and that the doctor said he could order nothing else, as he was bound by red tape. She also complained that while she was on the "Assaye" she had to tear up her apron and dress for bandages, although there was plenty of dressing on board. The allegations of Nurse Roberts were to some extent corroborated by an orderly from the "Assaye," who admitted that there was friction between the nurses and doctors, but declared that there was plenty of food. The civil surgeon on the "Lismore Castle" denied the allegations of Nurse Roberts *in toto*, and stated that she was dismissed as incompetent. On the other hand, an orderly from the "Assaye" said that a testimonial was given to her, signed by about 75 per cent. of the patients on board, thanking her for her kindness to them. According to Captain Holland, assistant naval transport officer, who fitted up the "Sumatra" before her voyage, and saw all the invalids taken on board, no complaint was made about any ship until Nurse Roberts gave her evidence. In the face of these conflicting statements, both nurses and the public will do well to refrain from forming a conclusion until the report of the Commission is published. It is really impossible to appraise the precise value of the evidence given by individual witnesses on the strength of the brief summaries cabled to London papers.

### RED-TAPEISM AT EUSTON.

A nursing sister from South Africa, on presenting her warrant for a railway pass, was told by the officials at Euston Station that they did not intend to recognise it. "That is a matter," she rejoined, "that must be settled between the railway company and the War Office; and" she added, "the sisters ranked as officers, and were provided with similar warrants." "No," retorted the officials, "we are not going to allow women to pass; warrants apply to officers, not to women." "Then," said the Sister in her natural indignation—while some eight clerks in the room dropped their pens to listen—"God help your poor brothers on the battle-field if the women refused to nurse them!" and she paid the full fare to Scotland.

### WANTED—WASHING UTENSILS.

One of the things that ought to be altered, says a Nursing Sister fresh from South Africa, is the very inadequate supply of washing utensils. In a marquee holding eight patients there was one basin, and this had to do duty for everything! Any nurse's imagination can doubtless fill in details. As our informant says, "It is a matter quite likely to pass unobserved by the mere man."

### THE STAFF OF THE "SIMLA."

The s.s. "Simla" has again arrived at Southampton with sick and wounded from South Africa. There were very few invalids, only about twenty being really ill, and even these had nearly recovered by the time the ship reached England. The Superintendent Sister was Sister Drury, of the Army Nursing Service. There were besides Sister Todd, A.N.S.; Sisters Lamondson and Sykes, of the Army Nursing Reserve; and three Civilian Nursing Sisters.

### GRIEVANCES IN INDIAN HOSPITALS.

It is not only respecting the Madras General Hospital that complaints as to nursing arrangements are rife. In a Bombay paper attention is drawn to the treatment of the Eurasian plague nurses. At the Jelarpet Junction Camp these nurses were receiving a salary of 100 rupees. This was first reduced to 50, and now it is proposed to further reduce it to 20. If exception is taken, natives are to be appointed at from 10 to 15 rupees. We are glad that a protest has been made against such shabby treatment of a deserving class. But there is, it seems, a hospital in the Bombay Presidency against which a more serious charge than false economy can be urged. At this institution, says a former plague nurse, certificates of training are given after one year's work, if the nurses pay for them. "On the other hand, if they are unable to pay they have to go through a curriculum of three years' training." The name of the hospital should, however, have been ascertained by our Anglo-Indian contemporary, in order that it may enjoy the odium it deserves, and that certificates emanating from it may be subject to the scrutiny they need.

### CALCUTTA GENERAL HOSPITAL.

A FEATURE of the new Presidency General Hospital at Calcutta will be the separate three-storied block capable of accommodating forty-six nurses and two sisters. It is to be erected on the site selected by the Superintendent of the Hospital, and Inspector-General of Civil Hospitals, Bengal, sufficient space being wisely left at the end for the future additions, if necessary, of another set of rooms. The building is to be provided with a flued floor 5 feet high, to keep the rooms of the ground floor dry. On the ground floor there will be ten rooms, nine of which will be occupied by eighteen nurses, and one in the centre will contain the main staircase, a verandah being provided to the full length of the building in front. A separate block is intended for bath-rooms connected to the main building by a covered passage. The arrangements on the first and second floors are similar to those on the ground floor, except that four rooms on the first floor are to be set apart as sitting-rooms and bed-rooms for two sisters in charge; two of these will have a verandah with bath-rooms at the rear built on cast-iron columns. Besides the main stairs, which will lead to the roof, there will be spiral staircases at the back for the servants. A covered way to connect the new building with the existing nurses' home, to enable the nurses occupying the former to have access to the latter building for meals, &c., in all weathers, is a part of the scheme.

### PROMOTION FROM WITHIN.

THE matron of the Royal Devon and Exeter Hospital states in an interview, reported in another column, that it is the custom at that institution to promote staff nurses to be sisters as vacancies arise. The present assistant matron and the night superintendent were formerly staff nurses. There is no doubt that it is sound policy to promote a certain proportion of the upper staff from within, providing always that the talent and experience available are all that could be desired. But a hard and fast rule would be a mistake. New blood is sometimes wanted in the nursing staff of a hospital, as elsewhere, and the matron at Exeter herself entered as a sister. Certainly in individual cases, if other things are equal, the inside candidate for an appointment should have the preference.

### THE QUEEN'S NURSES IN CORNWALL.

AN entertainment was given the other day by the Earl of Mount Edgcumbe to the Cornwall county nurses. In proposing the toast of "The Queen," Lord Mount Edgcumbe alluded to the progress made, and to the fact that there are now nine Queen's Nurses working in the county. He mentioned that Miss Peter, the general superintendent of the Nurses' Institute, had said that Cornwall was "A1" among all the counties in which associations had been formed, and carried out the rules better than any other. "It was a great advantage to young women in Cornwall," continued Lord Mount Edgcumbe, "to be able to obtain a year's training gratuitously. Of course, nurses in training were expected in return to serve the Association for three years, and it was hoped that many would remain longer. As time went on some districts would be able to offer higher salaries, and the best nurses, and those who had served longest, would probably be appointed to those districts, while some might be found qualified to receive further training, and themselves become Queen's Nurses."

### NURSES AND THE WORKMEN'S COMPENSATION ACT.

A LONDON doctor, whose identity is not disclosed, advocates the extension of the Workmen's Compensation Act to trained nurses. What do nurses themselves think of the proposal? There is, of course, no doubt that they often not only run the risk of a dangerous illness while they are on duty, but sometimes actually contract it. This is the peril of the profession, and the hard lot of those who succumb to it should, as far as possible, be systematically alleviated. In most hospitals, however, the utmost care and attention is already given to nurses who fall ill, and the special quarters assigned to such cases in some of the London institutions are all that could be desired. We are afraid that this is not always so. But the inclusion of nurses in the provisions of the Workmen's Compensation Act would certainly have the effect of making private persons less ready to employ them, and would inevitably lead to litigation. It might as well be urged that the Act should also be extended to doctors, who are exposed to exactly the same peril.

### AN IMPORTANT ISSUE.

ON July 2nd four probationers sent by the Technical Instruction Committee of the Cheshire County Council to the Nurses' Home at Plaistow commenced their duties, and at a meeting of the committee it was stated that claims had been received in respect of the outfit and six months' training fees at the home, amounting to £29 18s. 3d. for each nurse, with accounts for clothing and travelling expenses. It was resolved that the same be passed and recommended for payment. The Clerk then reported that in order that the question as to the maintenance of nurses at the expiration of their training might be understood and arranged for, he had addressed a communication on the subject to the Local Government Board, and had received a reply in the following terms: "I am directed by the Local Government Board to advert to your letter of the 7th ult., and in reply to state that the Board have no jurisdiction to determine the question therein submitted at the present time, but they are not aware of any provisions in the Technical Instruction Acts which would authorise the County Council to contribute towards the maintenance of nurses as suggested. I am to add that in the limited case of isolation hospitals provided under the Isolation Hospitals Act, 1893, some part of what the County Council have in view might perhaps be attained under the provisions of sections 17 and 21 of that Act." No action was taken by the Council in the matter.

### FOUR YEARS' TRAINING AT LAMBETH INFIRMARY.

THE Infirmary Committee of the Lambeth Board of Guardians have under consideration a proposal to require the nurses to serve one year at £35 after their probationary training of three years. If this proposal is carried into effect four years' training will practically be enforced. It seems to be the opinion of the Guardians that the constant departure of nurses to take up private work at better salaries may thus be checked. We doubt it, though the fact that the nurses of the Lambeth Infirmary are in great request for private work indicates that the training is considered satisfactory.

### ACCIDENTS TO CYCLISTS.

Two serious accidents to nurses while engaged in cycling are reported. One occurred at Annahilt to Miss Black, a nurse at the Lisburn Workhouse Infirmary, who ran into the shaft of a cart. According to the daily papers, the carman laid her in the ditch—where she was found an hour later insensible—and went away. He had not the gallantry of a typical Irishman, who would certainly not have left her until she had regained consciousness. On Saturday Miss Colley, a nurse at the Samaritan Free Hospital, Marylebone Road, collided with a cab, and was seriously bruised. She is, however, now going on well. We are very sorry for both nurses, but their misfortunes suggest that sufficient care is not taken to guard against mishaps. Cycling is not only a splendid and convenient form of recreation for nurses; it is also often indispensable in the pursuit of professional duties. This renders it all the more desirable that no prejudice should be excited against it owing to any want of prudence on the part of the riders, whether in the busy streets of London or less thronged thoroughfares, where danger is not so readily perceived.

### A NURSING HOME IN DIFFICULTIES.

The proprietor of a nursing home at Torquay has been summoned in the county court, and a committal order, suspended for 28 days, has been made against her. It appears from the evidence that at one time she had twelve or fourteen patients, and employed three or four nurses. Now she has no patients and only one nurse. According to her own statements, which include the admission that all her furniture, except a few little valueless articles, have been had on the hire system, she started the venture hoping to pay her way as she went along. This, after a while, she found impossible, and her unfortunate embarrassments afford another illustration of the extreme folly of persons starting a nursing home without sufficient capital.

### PITLOCHRY DISTRICT NURSING HOME.

There is every reason to believe that the Pitlochry District Nursing Home will be ready for the reception of patients next summer. It will be a memorial to the late Dr. W. S. Irvine, of Craigatin, and it has just been decided to proceed with it owing to an unexpected gift of £100, which, with the sum of between £700 and £800 in hand, led the committee to agree upon the adoption of a suitable design. The expenditure of about £1,000 has been determined upon, but this does not include painting, laying out the grounds, boundary walls, and furniture for the home. The latter will be a plain harled structure, containing two wards, male and female, with residence for the district nurse, and suitable offices. Miss Jane Cowan, of Logreach, has signified her readiness to support one bed in the home in perpetuity, and it is thought that others may come forward, and join in this manner in contributing to the memorial of a physician whose popularity in Pitlochry was as general as it was well-deserved.

### LINCOLN WORKHOUSE.

An inspector of the Local Government Board has been to Lincoln to urge upon the Guardians the necessity of increasing the nursing staff. He has made out an unanswerable case. The average number of patients is about 85, practically half of whom are bedridden. To attend to these are four nurses—a superintendent nurse, a day nurse on the male side, a day nurse on the female side, and one night nurse. This, as the inspector pointed out, means that during the day each nurse has to look after 40 breakfasts, and attend to all the wants of 40 invalids, 20 of whom are bedridden; whilst at night there is only one nurse for 80. The recommendation to improve these arrangements, which are obviously defective, is under the consideration of the House Committee, and we presume that it will be acted upon without delay.

### DISTRICT NURSING IN THE ISLE OF WIGHT.

The members of the Newport Nursing Society must be congratulated upon a financial state of affairs which shows that the work of district nurses in the Isle of Wight is appreciated. The report which was submitted at the annual meeting the other day disclosed the fact that the society has in hand a balance of nearly £50. During the year 70 persons were nursed and 2,850 visits were paid. Included in the sum raised was £22 14s. (the proceeds of an entertainment), various offertories from the churches, a contribution from the Mayor's fund, and a donation from a Lodge of Oddfellows. The further increase of annual subscriptions is still asked for. These should always constitute the bulk of the income of a nursing society, instead of a small proportion, as is too often the case.

### THE "DECAYED GENTLEWOMAN."

Nurses may be interested to know that an association has been formed to teach that sad survival, the "decayed gentlewoman," how to make fondants, nougat, and other dainties, beloved by the sweet, and sound, tooth of childhood. These associations are rather pathetic things, and have a side which is good and one which is evil, or at least foolish. If there are poor ladies who cling so fondly to their gentility that they will not work side by side with women less genteel than themselves, it is a good thing that they have friends generous and patient enough to set them up in such fashion as they will accept, and provide them with useful work. And if they do as good work as their ungenteel rivals in the trade, it is all right, and presently they will be decayed gentlewomen no longer but self-supporting and flourishing ones. But if they do not, they and their association must inevitably go to the wall. For a time, friends may purchase their wares to encourage them; but one's friends soon tire of that, and very few people have a social circle large enough to maintain them in this way. In the long run their work must appeal to the public at large, which neither knows nor cares whether their cigarettes are rolled and their nougat made by ladies or not. It is quality, not sentiment, the public cares for, and unless that is satisfactory, the wares will not hold the market long.

### SHORT ITEMS.

Miss Edith M. Wilson, who left England on June 2nd, writes to us from No. 7 General Hospital, Estcourt, that she was trained at the Leicester Infirmary for three years, and prior to her departure for South Africa was engaged in private nursing at Folkestone.

### Lectures on Nursing for Probationers.

By E. MacDowel Cosgrave, M.D., &c., Lecturer to the Dublin Metropolitan Technical School for Nurses.

### XVI.—AIR AND VENTILATION.

To be in a state of health it is necessary to have healthy blood, and this cannot be had unless pure air is breathed, for in crowded, ill-ventilated places the oxygen is not sufficiently absorbed, and the carbonic acid gas is not sufficiently given off, and impurities are breathed in and poison the blood. Breathing bad air causes debility, anæmia, and increased liability to disease; it also increases the death-rate, especially amongst children.

Some diseases, such as consumption, are much commoner and more fatal to those who suffer from a want of fresh air. This is clearly shown by army statistics; as new barracks, with larger air space and better ventilation, have been built, the death-rate from consumption in the army has rapidly fallen. It is easy to give the blood sufficient oxygen in the open air, for as each breath is given out it passes away and fresh air taking its place is drawn in. In buildings, however, the problem is not so easy, as if there is imperfect ventilation the air gets more and more contaminated, and gradually headache, lassitude, and other unpleasant symptoms appear. In order to prevent such symptoms there must be thorough ventilation.

Pure air contains in each 100 parts about—nitrogen, 80 parts; oxygen, 20 parts; carbonic acid gas, a very little (about 4 parts in 10,000). There is also more or less moisture, and possibly a trace of animal matter.

Oxygen is the active gas; it does not burn, but is a powerful supporter of combustion; if the still glowing end of a recently extinguished taper be plunged into pure oxygen it will at once rekindle. Oxygen is the only part of the air absorbed into the blood. Some of the air is in the form of ozone, which is a specially active condition of oxygen. Nitrogen may be looked upon as merely diluting the oxygen.

Carbonic acid gas is a very heavy gas, and checks combustion; if a tumbler full of carbonic acid gas be slowly poured out over a candle the gas will fall down and extinguish the light. When this gas is shaken up with lime water it joins with the lime, forming a milky mixture.

When air is breathed out it contains, in 100 parts, about—nitrogen, 80 parts; oxygen, 16 parts; carbonic acid gas, 4 parts. It is saturated with moisture, and is raised to the temperature of the body. Animal impurities are increased. The animal matters are ammonia, resulting from decay, and living microbes, of which many thousands to the cubic yard are often found in the air of crowded buildings. So in respiration, the nitrogen remains unchanged, one-fifth of the oxygen is lost, there is one hundred times as much carbonic acid gas, the moisture and impurities are increased, and the temperature is raised.

Were it not for plants, the oxygen of the air would long ago have been used up; but plants absorb the carbonic acid gas animals have breathed out and use it as food, giving the oxygen back once more to the air.

If the carbonic acid gas increases until it forms six parts in 10,000 (that is, half as much again as in pure air) it becomes injurious. There is an easy test to see whether the air of a building is contaminated beyond this permissible limit. An ounce of fresh lime water is put into a pint bottle of the air to be tested and is well shaken up; if there is less than six parts of carbonic acid gas in 10,000 of air the lime water will remain clear, if there is more the lime water will become milky.

When at rest about fifteen cubic feet of air pass through the lungs each hour. In exertion this amount may be doubled, but it has been found that to prevent the impurity of air getting above the permissible quantity in all cases at least 3,000 feet of air must be supplied for each person each hour. As it is impossible to change the air of a room oftener than three times in an hour without causing a draught, it is necessary that each person should have 1,000 cubic feet of space; for each sick person 1,500 feet should be allowed.

To measure the number of cubic feet in a room take the number of feet in the length, in the breadth, and in the height, and multiply all together. Thus a space 10 feet long, 10 feet wide, and 10 feet high contains 1,000 cubic feet of air. A room 35 by 20 by 13 contains 9,100 feet, and is large enough to hold nine healthy or six sick.

There are three forces in natural ventilation: (1) The breathed out air is warmer and lighter, and so ascends. (2) Diffusion, or the way in which gases mix, makes the expired air and the surrounding air change places. (3) The wind; this may blow air in, or draw it out, as it does for instance when blowing over a chimney. In artificial ventilation advantage is taken of these three processes.

Two sets of openings must be considered:—

(1) Outlets.—If the breathed air cannot be got rid of at once (as can easily be done in large buildings by having openings over the gas lights) it is best to have the outlets low down, for though when first breathed out the hot breath rises, as it cools the weight of the carbonic acid gas causes it to gradually fall to the lower part of the room; the fireplace is therefore one of the best outlets, and it is important to see that the chimney is not blocked. Even a small fire greatly increases the usefulness of the chimney by causing an up draught; it is generally considered that a fire will ventilate a room holding from three to six people. The top of the window often makes a useful outlet, and special openings for the same purpose are often placed near the ceiling. When gas is burned it is very important to have an opening over the burner to carry away the products of combustion, as gas uses up a great deal of air.

(2) Inlets.—Pure air must be admitted to take the place of that removed through the outlets. Some people do not realise this necessity, and try to exclude air by sandbags on the windows, india-rubber round the doors, &c. But if air goes out air must come in to take its place, and if every other inlet is closed air will come down the chimney and smoke will be puffed into the room. A good way to test whether the inlets are sufficient is to hold a lighted candle to the keyhole; if air rushes in and the flame is blown aside it shows that the proper inlets are not provided, for air will not force its way through the keyhole if an easier mode of entry can be found, as is seen when the window is opened and the chimney ceases to smoke. Air, if properly directed, may be let in freely; it is when it blows straight in without mixing with the warm air in the room that it causes the feeling of draught. The upper part of the window is the best inlet; when the upper sash falls inwards the current of air is directed to the ceiling, and no draught is felt. When it opens in the ordinary manner a blind or curtain should be arranged so as to break up the current of air, or a screen may be placed between the bed and the window. Placing a plank edgeways under the lower sash allows air to pass upwards between the sashes; this may usually be done with advantage in sick rooms. Tobin's tube is a commonly used and very good inlet. It takes in air at the level of the floor, discharging it into the room some four or five feet above the ground; as the air has an upward tendency it gets mixed with the air of the room, and does not cause a draught.

It is often a good plan, especially in the morning after the room has been done up, to cover the patient warmly up and throw open the windows, allowing thorough ventilation. A sick room should be ventilated by the window, and not by the door, especially at night-time when the staircase windows are closed, for if there are leaky drains the air of the staircase will be contaminated. If the door has to be kept open a lobby window must also be kept open.

The feeling of draught depends upon three things: (1) Coldness of air; (2) rapidity of its movement; (3) direction in which it moves. By enlarging the opening the rapidity can be lessened. Thus draught may be felt when a window is only an inch up, whilst if the window is opened widely the air can enter slowly and no draught is felt. By directing the air away from the inmates, and allowing it to mix with the air of the room, the first and third causes are avoided.

### The Nurses of the Royal Devon and Exeter Hospital.

### A CHAT WITH THE MATRON.

By Our Commissioner.

There is no difficulty in finding the Devon and Exeter Hospital. The cathedral is in the centre of the city, and the hospital is within a very short distance of the cathedral. It stands well, and the views of the surrounding country from the upper stories are picturesque. On my arrival, the matron, who confessed that she takes a pride in showing visitors over the building, conducted me into the wards of the Halford wing, in which are situated the male wards. As we proceeded Miss Bath pointed out many features of special interest. The hospital has undergone extensive alterations during the last few years, the main feature of which is the addition of the Victoria wing, which is set apart for female patients. Among the many recent improvements may be mentioned the kitchen, which now occupies the site of an old ward on an upper story instead of being in the basement as formerly. The hospital now contains five large large and handsome male wards, four female wards, and small separation wards attached to each ward, also a child's ward, containing twelve pretty cots. I noticed that in all the wards, which were bright with flowers of varying hues, there was an organ.

"The organs," said the matron, "were the gift of Miss Bickersteth, daughter of the Bishop of Exeter, and other ladies. Miss Bickersteth also supplies the books for a lending library, and changes them every week. We are entirely indebted to the kind energy of the late Lieut.-Colonel Lucas (then president) for the visit of T.R.H. the Duke and Duchess of York in July last year, when purses to the amount of £600 were presented to the Duchess of York. After the Royal visit the President then obtained permission from the Queen for our good hospital to be called Royal."

There is a well-appointed chapel, seating a hundred people, in which service takes place daily and twice on Sunday, the chaplain being resident in a house in the grounds. The Sisters' rooms, which are away from the wards, are tastefully fitted up. Another recent improvement is the installation of the electric light throughout the hospital, the same being generated on the premises. The grounds, which we walked through to the laundry, are singularly pleasant, and include tennis and croquet lawns. Ample accommodation is also provided for cycles. The laundry is fitted with powerful engines and modern machinery.

### The Steam Laundry.

"How long have you had a laundry?" I inquired.

"For about four years. There is a material saving in expense; but the laundry considerably adds to the matron's responsibility. I may say that every precaution is taken to dry the linen. It is all sorted and put in separate cubicles, each nurse having her own number. Hot-water pipes are laid on in the clean linen-room, so that it is always warm. There are also clean linen rooms attached to each ward heated with hot air, so that the ward linen is always well aired."

Then we explored the nurses' home, which is approached from the hospital by a covered way.

"Has there always been a covered way?" I continued.

"No; but we found that it was very badly wanted. It has been a great boon to the nurses, and I am sure often saves them in inclement weather from taking cold."

### The Nurses' Home.

"The nurses must also appreciate this beautiful recreation room," I said, as I glanced round that commodious and cheerful apartment before inspecting the library and the sleeping-rooms.

"There is sleeping accommodation for thirty nurses in the home," resumed the matron, "and, as you see, separate rooms for staff nurses. Our urgent want is more sleeping accommodation in the nurses' home."

"How many nurses are there in the hospital?"

"Eighty, including the private staff, of whom there are thirty."

"Where do they nurse?"

"We send them wherever they are wanted. Most of them are trained here."

### Four Years' Training.

"And the training?"

"The period is four years."

"Do you find it work well?"

"I think that it will. Obviously more experience can be gained in four years than in three. If the probationers pass examinations successfully they are made staff nurses at the end of the first year."

"What are the hours off duty?"

### Probationers and Operations.

"Sisters and staff nurses have three hours every day off duty, and probationers two. Each nurse is off duty every alternate Sunday. We have just started a new arrangement, by which all probationers are on duty in the morning, and are thus able to witness all the operations, and they take their off-duty time in the afternoon."

"How does the new arrangement answer?"

"We find it answers well. The probationers have the opportunity of seeing and learning how to do the most important dressings. Each member of the staff has a day off every month, and if private nurses are engaged for two months on a case they get two days subsequently. As to holidays, the sisters and private nurses have a month, the staff nurses three weeks, and the probationers a fortnight the first year and three weeks afterwards."

### The Instruction.

"As to the course of instruction?"

"Lectures go on through the year by two surgeons and two physicians. The house surgeon also gives clinical lectures. The theatre sister conducts instrument classes once a week, and I take the practical classes twice a week. In October we are going to have an outside examiner. We are very anxious that this should be a good training school."

"At what age do you receive probationers?"

"From 23 to 35. We have plenty of applications, but they are all very carefully considered, with the view of keeping the standard as high as possible."

"What is the number of sisters?"

"Nine. There is also an assistant matron and a night superintendent. In the large wards there are 35 beds; and in each of these, in addition to the sister in charge, there are a staff nurse, three probationers, and a night nurse. We promote our nurses to be sisters as vacancies arise."

### Promotion from Within.

"Does the practice of promoting from within extend to the assistant matron and the night superintendent?"

"Yes, both were formerly staff nurses. I think that it is a great thing to promote the staff. It acts as a kind of stimulus on the nurses when they know that they rise by merit."

### Recent Alterations.

"Have you made any alterations in the rules and regulations?"

"In addition to the alterations in the hours off duty, we do not allow a probationer to be placed on night duty until she has had six or nine, instead of three, months' training. The salary to nurses on the private staff, which formerly increased £2 10s. per annum with a maximum of £30, now increases £5 with a maximum of £40, and £5 per annum uniform money is given. Aprons, collars, and cuffs are now provided for the first year, as well as dresses and caps."

RECREATIONS AND PENSIONS.

"Do you care to say anything about recreations?"

"The hours off duty are convenient for cycling. For instance, the staff nurses go off at six p.m., and do not come on until the next morning. Tennis and croquet are provided, but croquet seems to have lost its charm. I allow the nurses to go to concerts and the theatre. They require recreation and change. Connected with our hospital we have a convalescent home, called 'Ockenden,' at Torquay, which was presented to us by Miss Erle, a resident of Torquay. This home is an immense benefit to our patients, and the nurses love going there for their days off. Other patients are also taken there from all parts."

"Is there any scheme of pension in operation?"

"A great number of sisters and nurses are members of the Royal National Pension Fund, but we are not rich enough to render assistance."

And then, congratulating the matron on the beautiful up-to-date hospital which Exeter possesses, I took my leave.

### Working in the Famine Districts of India.

BY A NURSE AT A STATION HOSPITAL.

OF the sufferings from famine I can give but a very faint idea. One little baby was brought in from an outlying district by the police; it had the appearance of a monster pin-cushion. Apparently, the mother had thought that on the top of a cactus hedge the baby would have a better chance of being seen than if she put it on the ground; doubtless she herself had returned to the village and died. With the aid of forceps, the thorns, or some of them, were extracted, and after a nice warm bath and bottle, baby was quite as well as could be expected; a number of festering sores resulted, but healed wonderfully well. The "crier" went round several of the villages adjoining the one near which the baby had been found, and a number of men came to see whether they could identify the child, but no one claimed it. Eventually, the Municipality undertook to provide for the little thing in some way. The idea of the men coming to "view" the baby may seem strange. But it arises in this way. Many of the men leave their native villages in this sad time and set off to try and find employment in some other district, and if successful return to their homes to take back their families with them. Then, in instances such as I have mentioned, the village "Patel," or headman, will give them all the information he possesses respecting the fate of the absent ones. From villages near us the poor had flocked, and the number of beggars on every road made walking all but an impossibility.

PATIENTS FROM THE MISSION STATIONS.

At the mission stations they do all they can. For example, three little boys rescued by one of the missions have more or less lost their eyesight through the general bad state of health arising from the prolonged starvation. They were brought to the hospital, but the medical officer told the missionary that we could do very little; careful feeding was the chief hope; and improvement in general health. However, they were admitted. To judge from the appearance of the boys, the dieting had been carried out "not wisely but too well"; the fact that the digestive organs were much impaired had apparently been overlooked. Their bodies, too, were covered with a most obstinate skin disease resembling "scabies." One child will only see dimly, another has recovered, almost entirely, the sight of one eye; in the third case one eye had to be excised, and the vision of the remaining one is almost perfect.

A SAD STORY.

One afternoon a girl, apparently about ten years old, looking very ill, walked into the hall, carrying a baby about nine months old; she begged for admission for the baby at least; there was no help for it but to take in both of them. After a rest she told us her story. Her mother had died of starvation, her last injunction to Dwarka being to put forth every effort to reach our hospital with the baby; she had once been a patient here herself. The child had begged her way along, sometimes obtaining a little milk for the baby, but very far from anything like a sufficiency for either of them. All Dwarka's struggle on baby's behalf was in vain; the little mite died in five days' time. Dwarka was heart-broken at first. After keeping her under observation for fourteen days and finding that no ulcers or abscesses made their appearance (too often these carried off the cases rescued from starvation), Dwarka was transferred to the convent of the sisterhood. After inquiries had been made as to any existing relations, and none being discovered, She was prepared for baptism. She is now a happy inmate of the home.

SEVERE CASES.

The women brought to us are often such skeletons that it is a matter of surprise how they live from minute to minute. Those reduced to this state are very difficult to feed, and many die. Most of the adult cases are admitted into isolation wards; one of the D.T. wards, too, being put into requisition, for in all severe cases of famine there is a very offensive odour from the body.

THE MEDICINE DIFFICULTY.

A number of girls, from seven to nineteen years of age, were at one time patients of mine. They were suffering from abscesses and scabies, the abscesses in almost every case being in the neck. A great difficulty was to prevail on them to take their medicines. I was obliged to give all medicines, excepting P.C.'s, immediately before they had their meals. The ward-woman brought their diets, and put each in its separate "thali" (plate) at a safe distance from the patients. Then I walked along the line thus formed with the medicines; no medicine, no food, had to be the order of the day. Sometimes one or other of the girls was very troublesome; then, after waiting a considerable time, I told the ward-woman to take away the diet belonging to the refractory one. This move always procured me a signal victory. Poulticing or dressing was very disappointing work. No sooner did I go into another ward than off came all the dressings, &c. At last I thought of a plan, and determined to see how it would work. These patients would eat salt by the handful, so having laid in a good stock of this commodity, I withheld it for the day from whoever removed dressings, &c. This was effectual, only it was necessary to watch the disobedient girl for a time, for she would fly at the others, and a general scrimmage followed. With regard to the P.C.'s, a little fruit or sugar was a sufficient bribe. It was necessary to be very exact as to the division of all diets, &c., for if one had a grain more than another a quarrel was the result. I am pleased to be able to say that all this soon disappeared, and that I obtained full obedience without any need of bribes.

EARLY ARRIVALS.

In times of great distress a few people always come to us between 4.30 and 5.30 a.m., and occupy the benches in the outer hall of the hospital. They have in most cases (during times of plague and cholera) travelled by night from the villages and evaded the police, feeling, I suppose, that they will stand a better chance of justice from inspection by the hospital authorities. In some cases they travel the whole of two or three nights, hiding under bridges, &c., during the day; for in times when cholera and plague are prevalent, all suspicious cases are stopped. Needless to say, if any of our early morning visitors turned out to be veritable cases of either, and had travelled any distance, their chance of recovery was infinitesimal indeed.

### Plague Inspection.

In the first few months of the railway plague inspection, before the Government hospitals were erected, there have been double rows of patients on the infectious ward verandahs. It was a time and scene ever to be remembered. A family of six, who had been travelling, with all their household goods, would be brought in with the patients. One family of four, besides their numerous bundles, were accompanied by a very fierce dog; it was impossible to keep it out of the wards. Fortunately they turned out not to be plague cases. As regards railway plague inspection of Europeans, in my own case I can only say that it was very thoroughly carried out. I was on my way to Cashmere with a friend; she was very delicate, and at one of the stations the inspecting officer was not satisfied. She had no fever, but seemed generally below par, as indeed she was, and we were both going for a rest to recoup. At the first toll out of British India we were met with two postcards, which we had to sign and affix dates to, stating that we were in perfect health. This was some ten or twelve days after the inspection, for we had broken our journey twice since then. We presumed that the cards had been presented to all travellers until we ourselves passed through.

### Nursing at No. 6 General Hospital.

Last week a member of the Army Nursing Service Reserve sent us an account of her work at No. 6 General Hospital. The following is a communication from another member of the service attached to the hospital: After various false alarms we left Naauwport on July 25th, some of the sisters having gone on a few days previously. The hospital in Naauwpoort consists now of one hundred beds only. We were delighted to be really off for we had been packed since June 4th, one night having even to sleep upon the floor because our beds were ready for transit. We took plenty of food with us as there was no knowing how long we should be in the train, but to our grief we were bundled out at Bloemfontein and told we must go to one of the hospitals in the town till we could proceed. Six of us were put into a marquee; I, as senior of the party, was lucky enough to get a bedroom, which I shared with a nurse belonging to the hospital. However, next day we were ordered to proceed, and to procure plenty of food before we started. Then, having added considerably to our ample store, the order was cancelled, and we did not start for another five days. When at last we left Bloemfontein it was by hospital train. We were bidden to be at the station to start at four p.m. o'clock, and waited till ten before we were really off.

### Travelling in a Hospital Train.

A hospital train is bliss for sick and wounded, but it is the contrary for people who are well. To begin with, notwithstanding its beautiful cleanliness and good ventilation, there is a decided odour of sick people, which is not to be wondered at when one remembers that it had been conveying enteric and dysentery cases down country for months past. Secondly, there was nowhere to sit except in our bunks, which are just like ships' berths, one above another, whilst down the middle of the train is a narrow passage. Here we took seats on our rugs to eat our meals as best we could. We had rations as well as our own private supplies, and for luncheon and dinner most excellently-cooked meat from the kitchen.

### Primitive Washing Arrangements.

It was all extremely funny, and we certainly did our share of laughing, and wished our friends at home could see our primitive washing arrangements. An orderly brought us a pail of water, and we had a small tin bowl, which served as our washing basin in turn. We travelled very slowly and jerkily all night. The country, so much as we could see through the slips of glass, was bare and black, flat as a board, and no one about but parties of scouts, and here and there a few Kaffirs. The devastated farms, &c., told their own sad tale of war. Sometimes we stopped for over an hour, and then the train orderlies used to rush out and have a game at football.

### On the Road to Johannesburg.

When we got to Kronstadt we were told that we should not be allowed to proceed, because there were rumours of fighting going on fifteen miles ahead, and we stopped there some time. The next morning we were again assured we should remain stationary, when, quite suddenly, an order came to proceed in a quarter of an hour, and for once we really went at the time stated, leaving three unfortunate sisters behind. However, it is an ill wind which blows no one any good, for we got more space, and we were able to move about a little. The men guarding the line must have a rough time of it. Sometimes they have a few tents, but more frequently they live in sort of holes dug out of the ground, and covered with sacking or bits of zinc for roofs. We stopped the night at a place called Kopjes, as the train was not allowed to proceed after dark, and we were speedily surrounded by soldiers. We were only too glad to have them tell us some of their experiences, or to secure an old paper.

### Our Arrival.

We reached Johannesburg, where we now are, the following day. The first thing which struck us was the glory of the mimosa. We are now in commandeered houses, and hardly know what to make of carpets, wardrobes, electric light, and such-like luxuries. To have drawers in which to put away clothing, after eight months living in one's boxes, is a joy. Things are fearfully dear here. Biscuits are 4s. a pound—only I don't think there are any to be got just now—and everything in proportion; but many things are quite unobtainable. Half the shops are shut, but the hospital in the splendid grounds of the Wanderers' Club, is exceedingly well arranged and organised, and we have the electric light in the marquees. There are plenty of sisters for the work, and there are some very good surgical cases.

### Nurses Wanted in Western Australia.

In view of the number of nurses who will shortly be returning from South Africa to England, with the necessary result of increasing the competition in the profession, the following extract from the letter of a nurse writing to a correspondent will be read with interest: "I am doing very well out here, and there is plenty of work for English nurses, should you hear of any wanting to come to Western Australia."

### Pictures from Italy.

By an English Nurse.

When my husband complained of feeling intensely cold I was not very much alarmed, though the temperature had been 90 deg. in the shade that day. On the shores of the Adriatic the breeze comes in from the sea at sunset and cools the air with a chilly rapidity. When, however, I found that the clinical thermometer of my nursing days gave him a temperature of 104 deg., and that a good dose of quinine had no effect, I began to be anxious. We were staying in a little town on the banks of the Tronto—a hundred miles, perhaps, from an English doctor. The little hotel in which we were staying was not particularly clean, and though the people were very kind it was not the most comfortable place to be ill in. The fact that about a dozen days before we had stayed at a little village in which—as we afterwards found—many of the inhabitants were down, typhoid, owing to some confusion between drains and waterworks, kept recurring to my mind. It was absolutely necessary to get a medical opinion. In Italy one always goes to the chemist's to find the doctor. In towns where there are large pharmacies he has a little room at the side, where he sees and prescribes for his patients. In small places he sits on a bench in the shop and takes a hand at cards or draughts with anyone who has leisure. When a patient comes for advice, everybody takes a share in the consultation, and helps both doctor and the afflicted by referring to similar cases they have known.

### "A Good Fever Doctor."

I left my invalid in bed and went to the only large chemist's shop in the town to make observations. In a corner of the shop, playing draughts with an old priest, was a dull-eyed man with imbecility written on every lineament. I made a purchase at the counter, and asked casually if there was a good fever doctor in the town. "Si, Signora; there he is." He pointed to the fat imbecile playing draughts with the priest. I left the shop with a heavy heart. Many years of nursing under some of the best English doctors had perhaps made me fastidious. I felt that in a long and possibly serious illness it was necessary to have perfect confidence in the medical attendant. My husband was even more sensitive than myself, and would have shrunk from the touch of those dirty fingers I had seen handling the draughts. I went back miserably to the hotel and wept on the bosom of the padrona, who, good kind soul, afterwards sent her whole establishment hunting the town for green figs and smoked ham, which was her idea of a dainty and comforting dish for my supper.

### Taking the Temperature every Ten Minutes.

Upstairs my invalid was hot and drowsy as ever. We tried the effect of anti-pyrin, and I got so terribly anxious that I took the temperature every ten minutes for the rest of the day. The poor patient suggested at last that I might leave the thermometer in his mouth altogether to save trouble. By the evening I was more convinced than ever before that neither mother nor wife should nurse her own dear ones. In the evening I left the boots to listen for my husband's bell, and went out to get some air. There was a green hill rising to the left of the town, and I scrambled to the top where one could look down on the quaint little town and the river in its tortuous ravine at the bottom of the valley.

### Looking for Lodgings.

The air was so sweet and fresh up there that I began to look round to see if there might be a house somewhere near in which I might obtain lodgings for my invalid. There was only one building, a square grey convent with a garden in front and a church tower rising from the farther corner. I do not know what instinct led me to peer through the gates, but at the first look it struck me that this convent no longer harboured brown-frocked monks or pale-faced nuns, but had been converted into a hospital for the sick. There was, moreover, an air of well-keptness about the place which gave the impression that it was a well-managed and up-to-date institution. I went up the broad path to the big door and found the porter, who led me upstairs to treat with the authorities.

### Finding a Surgeon.

In one of the cool long corridors a fair-haired young man in a linen suit—a typical house surgeon—was giving directions to an infermièra in brown holland. My heart leapt at the sight of his friendly, sensible face, and I began my business straight off.

"Signor dottore, can you take in a paying patient here—an Englishman?"

"What is the matter with him, Signora," he asked.

"I am afraid it is typhoid—we have been staying at a village near here where they have an outbreak."

"Yes; yes! I think I know the place. We have sent some of our infirmière to help them."

"Can you take my husband in, Signor dottore; and can you take me in to nurse him?"

"That is against our rules, Signora. We do not like the friends of patients to stay in the hospital. But we will ask the sister-superior if it is possible."

He took me into a room looking into the inner cortile of the convent.

### Willing to Take my Husband.

Seated at a desk was a lady in nun's costume with a refined and sympathetic face. She was quite willing to take in my husband, but did not want me.

"It is against the rules," she repeated, but very kindly.

"Yes, I know it is against rules, S'nora, but I am a trained English nurse, and can help in the hospital—you are short of nurses."

"But you are not accustomed to our ways—your English system is different."

"I have been taught to follow directions, and not to exceed them. Do take us in, S'nora, we are quite friendless here." Tears of disappointment were welling into my eyes.

The superior considered a little and looked at the house surgeon; he was evidently on my side.

"Well, my dear, perhaps we can manage it. But first the doctor will go to see your husband; he may not be as ill as you think," she said at last.

"By the way, have you consulted any other medical man?" said the doctor.

I was glad to say I had not. In Italy, as in other countries, patients sometimes die while questions of medical etiquette are being discussed. It was finally arranged that the doctor should come to the hotel to see the patient as soon as he had finished his evening round, and I went away much comforted.

### In the Hospital.

When the doctor came the patient took to him at once. He could not say whether the illness was typhoid, but agreed with me that the hospital was much better for an invalid than the hot, noisy hotel, and made every arrangement for our removal. By ten p.m. we were established in a large, airy room, looking out on the cortile of the hospital. A little bed was placed for me in a kind of ante-room, and everything was done for our comfort.

In the morning the temperature was down to 90 deg., and my invalid consequently in good spirits. The doctor found time to take me round the hospital, which was beautifully clean and well arranged. The operating-room, with its instruments, was in the most antiseptic condition, and the sister who presided over it appeared thoroughly grounded in her work. She was dressing a festered limb as we went through, and handled it in the most skilful way.

The patients had a generous allowance of the usual diet, but I found that the "brodo," which takes the place of English beef-tea, was flavoured with some herb, possibly rosemary, and that my husband would nottake it. I experienced no difficulty, however, in obtaining permission to superintend the preparation of beef tea and other things I wanted. The cook did my marketing and was my great friend. He would appear at the door of the sick room with a bowl of milk in one hand and a branch of sweet-smelling lemon-plant in the other, and many inquiries as to what sort of a night the signorina had passed. He had no business in that part of the hospital, but everybody was curious as to the ways and looks of the forestière.

After a day or two we were much relieved to find that the illness was not typhoid, and that a week's nursing would put the invalid on his feet. We stayed about ten days in the hospital and found it a pleasant place, which we left with regret and with thankful hearts towards the kindly authorities who had stretched their rules to take in the strangers.

### Nursing on the "Assaye."

### A TALK WITH ONE OF THE SISTERS.

By a Correspondent.

The main point of interest about the nursing on the "Assaye" was the very large amount of massage administered in cases of convalescence from wounds and fever.

"I was in charge of the officers," said Sister Millar, "with the very able assistance of Sister Bruce. Our work consisted chiefly of massage, and we found it most valuable in cases of stiffened limbs resulting from gunshot wounds, enteric fever, and rheumatism."

"Would you say that massage should form part of a nurse's training?" I inquired.

"I would certainly say that it is of very great use to a nurse," was the reply; "and I think it would be a very good thing if every ship were to carry a trained masseuse. I will give you a few instances on this particular voyage." And Sister Millar referred to the list of officers on board, and ran quickly through the names of those under her personal care.

### Some Cases.

"One officer was shot through the shoulder, and lost the use of his left arm; he had also had enteric; he improved very much on the voyage. Here is a case of arthritis with suppuration; half a dozen incisions had been made, the last one healed during the voyage; massage proved most valuable. The next was an officer said to be the youngest major in the British army; he had seven gunshot wounds, and suffered from almost complete loss of power in one arm. Another was a case of malaria, very serious, and here are three more with gunshot wounds; one had considerable stiffening of the wrist through a wound, another was wounded in the foot. In most of these cases we gave massage, and with very beneficial results. Another officer who had four wounds, in the shoulder, arm, and both ankles, was very seriously ill, and Sister Davison had him entirely under her care throughout the voyage."

Sister Millar then told me of a young officer who came from India who was so anxious to get to the front that he smuggled his horses and himself on board a transport, where he lived three days without being discovered. He had his wish, was given a commission, caught enteric fever, and afterwards developed rheumatic fever. He suffered from terrible stiffening of the joints, and was carried on board the "Assaye" in a perfectly helpless condition. He improved, however, every day of the voyage.

"They were so grateful for everything," Sister Millar went on, "and everything went so smoothly, there was not a single hitch throughout the voyage, and as there were 1,300 people on board, this is saying a good deal. When I came over in January the officers had, of course, seen very little active service, and no doubt they were disappointed; this time they had been through such terrible privations and sufferings that they appreciated the smallest comforts. One poor boy had lived for three days on one biscuit, standing up to his knees in water; and they had all had a hard time. If we had not been able to give them massage on the voyage, it would have meant, in many cases, operations under chloroform before the joints could be restored to their normal condition."

### Presents for Tommy.

"Had you anything to do with the privates?" I asked.

"Only in giving out tobacco and clothing. The things were brought on board by Professor Dunlop, and were mostly given by the ladies of Coatbridge and Airdrie, in Scotland. We were all so fond of Professor Dunlop, and so sorry when he had a very bad attack of bronchial catarrh during the voyage. He had been in charge of Wynberg Hospital, where he did very excellent work; he was coming home for rest, and is now, I am glad to say, quite well."

### The Sisters.

"How many nurses were in charge of the men?" I asked.

"Three," said Miss Millar. "Sister Goode and Sister Owen, who were fully trained, and Sister Duxbury, who did remarkably well, and was much liked. The others, whose names appear in this list of nursing sisters, were invalids, with the exception of Miss Vansittart, who was an indulgence passenger, on account of the excellent work she did at Kimberley, where she was linen-maid."

"How was the nursing arranged as to hours?"

"We breakfasted at nine—a very awkward hour, as we could not go on duty till half-past nine. Among the men duty began at ten, that being the doctors' hour. Lunch was at one, and sometimes we were able to get a little rest in the afternoon; then there were two or three massage cases, and a few more in the evening. Sister Goode did some massage for the men."

"And about night duty?"

"There was no necessity for the sisters to take night duty, as none of the cases were serious enough. It was sufficient if the orderlies called the doctors or nurses if necessity arose. The men were mostly recovering from enteric."

### The Medical Staff.

"We had a very excellent P.M.O.," Sister Millar continued, "in the person of Colonel Hodson. He was ably assisted by the ship's doctor, Dr. Harvey, who was very much liked. There was also a third, Civil Surgeon Steele, who was returning home after an attack of enteric in the Civil Hospital at Kimberley, where he was so ill that he was hardly expected to recover. He assisted the other two doctors."

### A Unique Position.

"We were in the unique position of having every corps in the British Army represented except eight. The exceptions were the 1st Royal Dragoons, the 13th, 18th, 19th, and 20th Hussars, the Devonshire Regiment, the Inniskilling Fusiliers, and the York and Lancaster Regiment."

### Praise Where it is Due.

Sister Millar was anxious that everyone should have their due meed of praise, and wished to make special mention of Sister Bruce's work at Modder River. She had seen mention made of the work of the Army Sisters there, and would like the public to know that Sister Bruce, who is a civil nurse, also did excellent work under great difficulties.

"She was there from the middle of February to the end of May, working hard, under awful conditions of privation and hardship," she said, "and I know she continued, even when her temperature was over 100."

### Personal Experiences.

Later, Sister Millar gave me some account of her own experiences of the war.

"I was at the Cape for my health last October," she said, "never dreaming that this terrible war would break out; then I found that I could not get up country or move about anywhere, and as soon as I was fit I offered myself for active service. I came home in the 'Canada' in January with sick and wounded, and found orders for me to join No. Eleven General Hospital. From March 3rd to May 13th I was at Orange River, attached to No. Eleven. In May and June I was nursing in the Masonic Hall at Kimberley, and a very excellent nursing home it made; nearly all our patients ecovered."

"Was there any understaffing?" I asked.

"Not after the first month, and that was because the cases were very acute, and came in in such numbers; this was at Orange River. It was pretty rough, indeed, and the men were dying in such numbers round us, for the fever was just at its height then."

### Sidelights.

Finally, I learn that Sister Millar, who is "a matron at home," as she said, got her training at the Glasgow Western Infirmary, and in a Glasgow paper she showed me an article which she had written in answer to Lady Sykes' book on "Sidelights on the Nursing in South Africa." In it she maintained the superiority of the trained nurse over the tyro, and gave her own experience of one of the "silly women" who was with difficulty prevented from giving a patient five drops of morphia without the doctor's permission; of gingerbread given to enteric patients; of undressed wounds during the voyage between Durban and Cape Town, at which port the trained sisters came on board; and of the joy of the patients when the untrained lady took her departure and the sister was in charge. Sister Millar also endorsed what other nurses have said, that the impossibility of transport by a single line of railway, in many places tampered with, together with the alarming rush of enteric fever, was the main cause of the complaints recently made against the provision for the nursing of the sick in South Africa.

### Everybody's Opinion.

[Correspondence on all subjects is invited, but we cannot in any way be responsible for the opinions expressed by our correspondents. No communication can be entertained if the name and address of the correspondent is not given, as a guarantee of good faith but not necessarily for publication, or unless one side of the paper only is written on.]

### MADRAS GENERAL HOSPITAL.

"One Who Knows" writes: Owing to absence from my head-quarters I have not seen The Hospital for some time, but on looking through the back numbers I find in your issue of July 28th a letter which interests me greatly. It is from "A Resident in Madras," and refers to the General Hospital in that city. I have heard much of the unsatisfactory state of that hospital, from the nurses' point of view, from a relation of my own who trained there. Judging by what she told me of her own experience (and hers was, she said, by no means an exceptional case) the condition of things is, indeed, as "A Resident in Madras" affirms, one which merits attention. I may add, what I know to be a fact, that many nurses who have left the hospital on account of the treatment they received, and who would gladly take advantage of the correspondence column of your valuable paper to warn others from a like fate, yet fear to do so as they are women who have to work in Madras, and the enmity of a certain powerful member of the nursing executive is a thing to be dreaded by them. Nurses now on the staff of the hospital are prevented from bringing the matter forward in the press by the terms of their Government service.

### NURSES AND MENIAL WORK.

"Alpha" writes: I was very much interested in reading the letter written by "F. R." with regard to the question, "Ought nurses to do so much menial work?" Now, while I think it quite essential that a nurse should have a knowledge of domestic duties, in order to fit her for imparting that knowledge to those who may require it at her hands, or if necessity compels her to perform those duties herself, I certainly do endorse "F. R.'s" contention that a nurse should not be subjected to anything menial as a nurse, beyond what occurs in her professional capacity. Not that it is a disgrace; on the contrary, domestic aptitude, I consider, is an essential of life more or less requisite in everyone under certain conditions. But it appears to me that the position of a professional nurse should place her above anything menial, as being of a professional standard, and consequently of a higher order in the social scale. I have the greatest admiration for the profession of nurse, as I think there is no calling in life more humane and noble than hers. For what is more humane than the ability and desire to alleviate pain and suffering? And what more noble than the desire to soothe the anguish and depression arising from pain, added to it, may be, the knowledge of being far from home and loved ones? Can there be anything more nearly realising the great ideal of the Good Samaritan? How many of our brave fellows have had cause—in South Africa—to repeat the act of gratitude as evinced lately by a working man, who, upon meeting a nurse, raised his hat, exclaiming, "God bless you, nurse!"

### NURSING HOMES IN SOUTH AFRICA.

"A Friend of Nurses" writes: At the present time, when the minds of all are directed towards South Africa and its future possibilities, it may be kind to give nurses a word of warning as to the relative value of money here and in that country. A friend of mine recently answered an oft-appearing advertisement for nurses for a home in a South African town, and upon application received particulars from which it appears that the salary offered is £60, board and lodging, washing, and medical attendance. Being entirely ignorant of the cost of living, my friend considered the salary a good one, and would probably have accepted an appointment had not an acquaintance with more experience enlightened her somewhat. The latter had been a nurse at Johannesburg, and found, when too late, that although she was paid at a higher rate than £60 a year, her salary did not go half as far as that amount would have done in England, and that it was very difficult to save. The work was as hard, and many nurses died of typhoid during the three months she was there. She had been given a first class passage out. The committee required the nurse to pay her own passage out, as well as railway expenses up country, and quoted the price of third class as well as second class on the steamer. I cannot understand a nursing home being even willing that their nurses, presumably ladies, or at any rate women of refinement, should travel third class to the Cape. Those who know the usual class of third-class passengers on the boats would sincerely pity the nurses, I think, although perhaps the "cabin reserved for them" by the lady who sends them out may do something to mitigate the discomforts. I note that should the nurses prove unsuitable on arrival, or their health break down, they will be given three months' notice. But unless they have private means how can they return to England? These and other points force themselves so strongly upon me that my advice to nurses going out to nursing homes in South Africa is, "Look well before you leap."

### Echoes from the Outside World.

### AN OPEN LETTER TO A HOSPITAL NURSE.

Talking to an Irish girl the other day, I was much amused to find that she looked upon it as a foregone conclusion that the Queen would visit Ireland again this spring. Her idea seemed to be that Her Majesty was so pleased with the Emerald Isle that she would naturally be unable to get through a whole year again without an Irish refreshener. I said nothing, because, of course, it is quite possible that the Queen may intend to honour Pat again. Meanwhile, I see that the proprietor of the Hotel Angst at Bordighera is already making preparations for the reception of the Queen in the early spring. He does not affirm that he has received direct Royal instructions, but appears to discreetly keep silence on the subject, whilst all the same he continues his arrangements, with the result that he is given credit for knowing a great deal more than he says. I think, however, it is probable that no one has any definite information on the subject, for a very good reason—that the Queen has not yet decided herself. So much depends upon circumstances.

---

The intended visit of the Duke and Duchess of York to Australia and New Zealand next spring has, of course, given much satisfaction across the seas. The Victorian Parliament has telegraphed an address of thanks, and so have the Governors of the other different colonies. Both Houses of the New Zealand Legislature have also adopted a resolution expressing pleasure at the Royal visit. An Australian lady, with more loyalty than commonsense, has suggested that one of the young princes, preferably Prince Edward of York, should accompany his parents, because all Australians would be so delighted to see him. But apart from the undesirability of two of the heirs to the throne being abroad at the same time, offering unprecedented opportunities to the horrid Anarchists, it would be the reverse of kind to a little boy of six to take him away from his brothers and sister, make him attend functions, always be on his best behaviour, and stop his education, which, as is invariably the case with Royal children, has begun in real earnest, even at his early age. It is bad enough for the chicks to be separated from their parents for so long, without making them suffer already the pains of being royally born. I see that according to Her Majesty's proclamation, January 1st is the date fixed for uniting the people of New South Wales, Victoria, Tasmania, South Australia, Western Australia, and Queensland into a Federal Commonwealth under the name of the Commonwealth of Australia.

---

It is five years since we had a General Election, and considering the advance which women have made during that time it is only natural to expect that they will probably play a more important part in assisting the return of members than they have done heretofore. As a sign of the times, I note that the Cardiff Women's Liberal Association are advertising for a lady organising secretary. Not that women have not always played a rôle in the electoral drama. Even decades ago the pretty wife of a candidate often secured his election by her pleasing manners, and I remember the wife of one of the members for Cambridgeshire, the possessor of a beautiful voice, singing to the electors, and so winning their hearts that they voted for her husband. At present the newest departure has been made by Mrs. Courtney, who has addressed a farewell message to Mr. Courtney's former constituents in the Bodmin division. "This," she says, "may be an unusual thing for a member's wife to do, but I cannot part without a word to the men and women who have been so good to me during the four elections that have taken place since a certain event in 1883 associated me with Mr. Courtney's fortunes. During two elections I was often in villages and at meetings by myself, and I cannot remember a single unkind or rude word addressed to me by the most eager opponents. I do remember some interesting and happy meetings, so for the rest of my life I shall carry about a very warm memory of the dwellers in south-east Cornwall. It is sad to part from them and to realise that there are many we shall not see again, so now it is good-bye and good wishes to all, especially to those who so faithfully followed my dear husband through so many changeful years." I should fancy that the Bodmin electors must personally be almost as sorry to lose Mrs. Courtney as she is to part from them. The other item of what I might call "female election news" is that the statement about the Lady Mayoress canvassing for her husband's candidature in West Southwark in the civic coach is absolutely false. I am glad it is. It would, to my mind, have been most unfair that because a man was Lord Mayor and had a glittering carriage for his official use he should have allowed it to be employed for electioneering purposes. There is, too, something so very impressive and overwhelming in the gorgeosity of the Lord Mayor's coach and the powdered flunkeys that I can imagine it carrying great weight with the very poor who seldom see much save costers' carts.

---

I have just heard of the following little incident, which seems to me so pathetic that I must tell you about it. An American lady is staying at Nordrach for the open-air cure for consumption, and with her is "her little daughter to bear her company." Lower down in the valley is a second little daughter, who is taken care of by another relative, it not being considered desirable that both children, who are aged respectively nine and seven, should be with their mother. Occasionally the latter goes down to see the younger child, and upon the last occasion she did not take the elder sister with her. This caused some grief, and in the evening, when she bade her good-bye, the little one said pathetically, "Oh, mother, I must see Joan. I want to see her so badly. I shall die if I don't!" The mother reproved the child for using such exaggerated language, and, comforting her as best she could, returned home and thought no more about it. Next evening some one at Nordrach, looking out from the windows, espied a weary-looking little figure coming slowly up the path. It was that of the seven-year-old maiden, who, slipping away from her relative soon after breakfast, had walked nearly eight miles across the country to get to her beloved sister! Of course there had been frequent rests by the wayside, but it was wonderful how the little stranger of such tender years in a foreign land had ever accomplished the journey at all. It shows what childish determination, fed by love, can do.

---

Few nurses have either time or inclination to read the electioneering speeches which are flooding the daily papers at the present time. But there is one undertaking, given by a very important member of the Government, which will, I think, afford real pleasure to every member of the profession. In reply to a series of questions at East Manchester on Monday, Mr. Balfour stated that Ministers intend to provide for the widows of all soldiers who die fighting for their country, and also for disabled soldiers themselves. The First Lord of the Treasury had not then the opportunity, and probably had not the power, to disclose the means by which such provision will be secured. It is, however, extremely satisfactory that he is able to pledge the Cabinet, if they remain in office, to take action.

### For Reading to the Sick.

S. MICHAEL AND ALL ANGELS.

ALL who, circling round, adore Thee,
All who bow before Thy throne,
Burn with flaming zeal before Thee,
Thy behest to carry down;
To and fro 'twixt earth and heaven
Speed they each on errands given.

Strong to aid the sick and dying,
Called from heaven they swiftly fly,
Grace Divine and strength supplying
In their mortal agony;
Souls released from bondage here
Safe to Paradise they bear.

—*Rev. W. Palmer (from the Latin).*

**Reading.**

"All angels, like all saints, occupy a festival day; unlike men, they give no cause for an introductory vigil." For sin it is which necessitates vigils, death, all else that is sorrowful. For sin is the only essentially grievous thing in the universe. God, Ever Blessed, had never that we can conceive known suffering if He had not "borne our sins in His own body on the tree." Good is human sorrow, for it makes men so far like Christ, who learnt sorrow for their sakes. The holy angels, who neither sin nor bear sins, know not sorrow. Even sympathy, one of our noblest sources of sorrow, is not, so far as we can tell, any source of sadness to them. They love us, yet cease not to rejoice; care for us, yet observe one unbroken jubilee. Their perfection hinders not their sympathy with us; the lack of sympathy is on our side, because also is the imperfection. . . . All is good which bears the stamp of a divine likeness. While life and joy cease not to be good, grief, death vigils have become likewise good, because Christ in His own Person has known them all.—*Christina Rossetti.*

Far in the glory of the sunset clouds,
Angels methinks are there;
But most where hearts—lone hearts—pale grief enshrouds,
They stand with radiant hair.

. . . . .

In solemn beauty, and in strength and power,
Comes the soul's guardian from his home afar
To stand beside us in temptation's hour,
Pure as a glittering star.

They teach us mysteries of life and death,
In the soul's silence breathing hallowed things;
With Heaven's hushed music in their fragrant breath,
God's glory on their wings.

. . . . .

A soul is wrestling! In Gethsemane
E'en to our Christ the holy angels came;
They waited on Him in His agony,
Shrouding in wings of flame.

And have we not need the faith to hold
Of the surrounding angel bands?

To feel them near in hours of toil and weeping,
With reverence hail each soul's celestial guest,
Till they shall come, the final harvest reaping,
To fold us into rest.

*E. Brine.*

If we could but have hearts to feel, and eyes in our souls to see where we really are! There are good angels round us, and graces are raining down upon us, great and small, all our lives long. Inspirations are falling thick as snow-flakes, and almost as softly and silently, and we are fastened with a thousand fastenings to great unknown purposes, and we feel them no more than a strong man feels the cobwebs and the gossamers in the autumnal grass. And all the while we are closed in, and walled round, not so much with the sun and moon and stars, with the air and floor of our own planet, as with the living and inevitable presence of the All Holy, who will not spare us one moment from His sight, and whose love and fears, and also His jealousy of us, is as everlasting as Himself.—*Faber.*

### An Incident in a Matron's Experience.

I WAS a young matron, and the responsibility of sixty delicate convalescent children from the slums of London and elsewhere weighed heavily upon me. How to influence for good these poor, neglected—often worse than motherless—little ones, and to make their stay with us a bright spot, to be remembered all their lives—these and other thoughts were on my mind as I took my place in the hall with nurse and children and household round me for morning prayers. The hymn went joyously, and the silence that followed as we knelt for prayers awed me, and I felt how reverent these poor neglected children were, and what an example they showed to children who had lived luxuriously, and who frequently are so restless at prayer time. Feeling this, and concluding the service, imagine my surprise when Nurse observed, "I am sorry, Matron, to have to report twelve boys for bad behaviour at prayers." "How, Nurse?" "They had two tiny crabs they got on the shore yesterday, and were urging them to race one another along the form in front of them." The boys knelt with their faces, not their backs, to us in future.

### Appointments.

THOMAS WALKER HOSPITAL, FRASERBURGH.—Miss Rachael C. Soutar has been appointed Nurse Matron. She was trained at the Sick Children's Hospital, Aberdeen, and she has since been staff nurse at Gray's Hospital, Elgin, charge nurse at Craiglockhart Hospital, Edinburgh, and on the staff of the Edinburgh Nurses' Association.

BRIGHTON AND HOVE HOSPITAL FOR WOMEN.—Miss M. H. Poulton has been appointed Matron. She was trained at the London Hospital, and has held in succession the post of superintendent at St. Mark's Hospital, matron at Woolwich and Plumstead Hospital, and matron at Boston Hospital.

LEWISHAM UNION INFIRMARY.—Miss Susanna Isabel Glanville has been appointed Matron. She was trained at St. Bartholomew's Hospital, London, and has since held the posts of assistant matron at Hackney Infirmary and matron of the Bromley and Beckenham Joint Hospital.

ST. SAVIOUR'S UNION INFIRMARY, SURREY.—Miss Ethel Gertrude Massey has been appointed Second Assistant Matron. She was trained at North Staffordshire Infirmary and Eye Hospital, and has since been sister at the Royal Infirmary, Derby, and sister at St. Saviour's Union Infirmary, East Dulwich, Surrey.

### To Nurses.

**WE invite contributions from any of our readers, and shall be glad to pay for "Notes on News from the Nursing World," or for articles describing nursing experiences, or dealing with any nursing question from an original point of view. The minimum payment for contributions is 5s., but we welcome interesting contributions of a column, or a page, in length. It may be added that notices of entertainments, presentations, and deaths are not paid for, but, of course, we are always glad to receive them. All rejected manuscripts are returned in due course, and all payments for manuscripts used are made as early as possible at the beginning of each quarter.**

### Notes and Queries.

**THE Editor is always willing to answer in this column, without any fee, all reasonable questions, as soon as possible.**

**But the following rules must be carefully observed :—**

**1. Every communication must be accompanied by the name and address of the writer.**

**2. The question must always bear upon nursing, directly or indirectly.**

**If an answer is required by letter a fee of half-a-crown must be enclosed with the note containing the inquiry.**

*Consumption.*

(255) Would you kindly tell me if there is any place where a consumptive child can have the open-air cure at a low charge? She belongs to very poor parents.—*H. W.*

Most of the consumption hospitals arrange for treating suitable cases on this plan. The North London Hospital for Consumption, Mount Vernon, Hampstead, N.W. (office, 41, Fitzroy Square), admits patients by governor's letter, or on the payment of a moderate fee. Also at the Sanatorium at Bournemouth.

*Maternity Nursing.*

(256) In 1896 I obtained a certificate for monthly nursing from Queen Charlotte's Hospital. Since then, and having heard and read so much about the difference between the experienced and inexperienced method of bathing a patient in bed, I should like to know how nurses with only maternity training find out the proper way; and also if it is considered detrimental to a lying-in patient to take a bath in bed?—*Nurse S.*

You touch upon a real difficulty; one which points to the desirability of all maternity nurses having been trained in sick nursing as well. If you joined the Midwives' Institute, 12, Buckingham Street, Strand, you would be helped in your difficulties of this kind. It is usual for patients to have a bath in bed unless it be forbidden by the doctor in attendance. If, however, you wash and dry your patient all over in "sections," so that she does not take cold, you will have given her an effectual blanket bath, though, perhaps, your performance may not be quite so skilful as that of a trained nurse.

*Nursing Enterprise.*

(257) Can you tell me if there is any opening for a nursing home at B. or C. B.? If not, can you suggest a place where a home is needed?—*Sister Lucy.*

It is obviously impossible for us to help our querist in this way. Too many nurses are ruined by such enterprises, and all that can be done is to urge those who contemplate such a plunge to look very well indeed before they leap.

*Australia.*

(258) Would you kindly tell me the best way, and to whom I should apply, for a post as matron, night superintendent, or sister in a foreign hospital? Australia or U.S.A. preferred.—*Florence.*

Write to the Emigration Office, 31, Broadway, Westminster, for the latest returns bearing upon nursing in the places you mention. Then consult the lists in "Burdett's Hospitals and Charities" of the more important public institutions and write to the superintendent of nurses of those suitable. If highly qualified you might get something to suit you through the Colonial Nursing Association, Imperial Institute, S.W.

*Naval Nurse.*

(259) Can you tell me how to become a naval nurse? I have a certificate from a general hospital, and several years' experience in private work.—*B. B.*

Apply the Director-General, Medical Department of the Navy, Admiralty, Craven House, Northumberland Avenue, W.C. The appointments in this service are few, vacancies rare, applications numerous, and foreign service obligatory.

*Phthisis.*

(260) Will anyone give me any information with regard to the Great Canary Isle as a health resort, or of any other equally suitable places for the early stage of phthisis?—*Mrs. C.*

It is the truest economy to consult a specialist about the case before taking the patient abroad. It requires great skill to select the locality best suited to a consumptive patient's idiosyncrasies, and the responsibility attached to a decision upon which depends the issues of life or death is very heavy.

*Nursing Lectures.*

(261) Will you kindly tell me if there is a society for publishing simple nursing lectures, suitable for villages and rural districts?—*District Nurse.*

There is no society especially for the purpose. The Scientific Press undertakes the publication of suitable books on nursing topics, and the manager would send you a list of such on application.

*Binding Covers.*

(262) Can you tell me where I can procure covers for binding a year's loose copies of THE HOSPITAL and "Nursing Mirror? How much will they cost?—*G. M. K.*

The covers are made for volumes composed of six months' numbers. The manager, the Scientific Press, will tell you all about them, and take your order.

*Edinburgh.*

(263) Can you give me the names of some homes for private nurses in Edinburgh? Is there a branch of the Nurses' Co-operation there?—*M. R.*

The Nurses' Co-operation has no branches. The Royal Scottish Nursing Association, 69, Queen Street, Edinburgh, is the largest.

*Four Probationers.*

(264) Six months ago we signed for a three years' training, but am very disappointed, as there is very little to learn, and this is not a recognised "training school.". We are anxious for a good training and certificate. Will you kindly tell us if it is possible for us to resign?—*Anxious.*

All depends on the exact wording of the agreement, and upon whether or no any misrepresentation was made in regard to the character of the training to be given. If there was no misrepresentation the probationers clearly must (so far as the law is concerned) abide by what they have signed. We fancy, however, that no wisely managed institution would care to be burdened for three years with four unwilling probationers working under a sense of injustice, and the best course will probably be to talk the matter over quietly with the clerk to the Board of Guardians, and see if some arrangement cannot be made for a release.

*Minor Operation.*

(265) The patient I am nursing has had all his upper teeth and all but four in his lower jaw extracted under ether. The question is, Does the administration of an anæsthetic make the difference between a "simple extraction" and a "minor operation"? The doctor attending the case considers that it does; the committee of the man's sick club have decided that it does not, and they have therefore only given him the allowance for the simple extraction.—*District Nurse.*

All depends upon the exact wording of the agreement between the club and its doctor. According to the ordinary meaning of words, a "simple extraction" *is* a "minor operation," and the very fact that a distinction is drawn shows that the words must be interpreted by the context in the agreement.

*Inoculation for Typhoid.*

(266) I am nursing in a typho-malarial region. Where can I find particulars of the lymph used to inoculate for prevention or ameliorating typhoid fever? I read of some of the South African nurses being so inoculated. Was it successful?—*Sister Helen.*

The whole question is at present *sub judice.* There seems reason, so far as statistics will take us, to believe that some protection has been given by inoculation. The lymph used came, we believe, from Netley.

*Revolving Huts.*

(267) Nurse W. would be greatly obliged if the editor could tell her where to obtain the "revolving huts" several times mentioned in this paper. Also for information as to probable cost, and as to the possibility of procuring a secondhand one.

Messrs. Boulton and Paul (Limited), Norwich.

*Redress.*

(268) Last February twelvemonths I received a kick from a delirious patient which resulted in internal injuries, incapacitating me from any work. Can I claim compensation? My patient sent me £2 last year, but will not give me further help.—*Nurse G. S.*

If the injury was received in the course of the ordinary duty of the nurse we believe that no compensation could be obtained unless it could be shown that there was some misrepresentation at the time of the engagement as to the nature of the case—in other words, as to the nature of the risk involved in accepting it.

*Ulceration of the Bowels.*

(269) 1. Can you tell me if ulceration of the bowels has ever been known to last for more than eighteen months? If so, how long? 2. Would you advise a woman over fifty to undergo an operation?—*J. H.*

1. Yes. 2. We cannot advise. Consult a doctor.

*Abroad.*

(270) Will you kindly give me the names of some nurses' homes abroad or tell me the best way to get work on the Continent for the winter?—*Nurse Maude.*

The English Nursing Home, Villa Albany, Biarritz; the Hollond Institute of English Trained Nurses, 25, Rue Amsterdam, Paris. The San Remo English Nurses' Institution has head-quarters in San Remo and branches in Bordighera and Alassio. The large nurses' associations are constantly sending nurses abroad.

*Advertising Agency.*

(271) Is there a reliable advertising agency which would undertake to insert an advertisement for a patient to go abroad in the various nursing and medical papers?—*Sister Norah.*

The usual agents undertake any special work required.

*Infirmary Training.*

(272) 1. Is an infirmary as good as a hospital training? 2. To what age are probationers taken? 3. When does the salary begin? 4. What is the shortest height acceptable?—*Louise P.*

1. Infirmaries are hospitals and hospitals are infirmaries as a general rule. The training is equally good in either. 2. 35. 3. It depends on the institution; in some it begins at once. 4. 5 ft.

**Standard Books of Reference.**

"The Nursing Profession: How and Where to Train." 2s. net; post free, 2s. 4d.

"The Nurses' Dictionary of Medical Terms." 2s.

"Burdett's Series of Nursing Text-Books." 1s. each.

"A Handbook for Nurses." (Illustrated.) 5s.

"Nursing: Its Theory and Practice." New Edition. 3s. 6d.

"Helps in Sickness and to Health." Fifteenth Thousand. 5s.

"The Physiological Feeding of Infants." 1s.

"The Physiological Nursery Chart." 1s., post free 1s. 3d.

All these are published by THE SCIENTIFIC PRESS, Ltd., and may be obtained through any bookseller or direct from the publishers, 28 & 29, Southampton Street, London, W.C.

### Travel Notes.

### LVIII.—ROUND ABOUT LUCERNE.

The great tendency with most people who have but little time at their disposal for a holiday is to be too ambitious, and to crowd too much into a fortnight or three weeks. Personally I think it is much better to explore thoroughly whatever locality one is placed in rather than to rush feverishly north, south, east, and west, getting perfunctory glimpses of dozens of places, but really studying none. It is thus with our Transatlantic cousins; it is marvellous what they contrive to see in a limited time, but at what cost of fatigue, what wear and tear of mind and body, and with what meagre results. I once met such a couple on the St. Gothard line; they could only be absent from New York two months. In that time, besides making the two voyages, they had visited Paris, Brussels, Cologne, Strassbourg, and Lucerne, and were going on to see in turn the Italian lakes, Milan, Venice, Florence, Rome, and Naples. They were already so tired that scenery was a burden, and the only thing that evoked any enthusiasm was our tea-making arrangements, which they pronounced to be "cunning." Seeing their enfeebled condition (they were elderly people) we gave them some of the cheering beverage, which revived their flagging energies and enabled them to contemplate with more serenity their approaching hurried glimpses of Como and Maggiore. The object of this digression is to advise you if you have not more than twenty-one days' holiday to spend it entirely in the immediate neighbourhood of Lucerne, and not to wander too far afield.

### The Ascent of Pilatus.

The ticket for the cog wheel railway is dearer than that on the Rigi line; the ascent costs ten francs and the descent six. There are certain trains in the early morning and late evening that give return tickets for twelve francs, but if you are tolerable walkers it is preferable to come down on foot. There are several ways of making the ascent; if you walk, the best point from which to start is Hergiswyl, on the Brunig railway, reached in a quarter of an hour from Lucerne. It will take from three and a-half to four hours' steady walking to reach the summit; but it is a perfectly easy road and absolutely safe if you do not wander from the beaten track. Another and easier way, though more prosaic, is to take the rail to Alpnach-Stad, there change to the mountain railway, which will bring you to Pilatus Kulm in an hour and a-half. There are two other ways for pedestrians, one from Alpnach-Stad by a bridle path and one from Kriens; this last is not to be undertaken without a guide unless you are experienced mountaineers. Kriens is reached from Lucerne by steam tram in a few minutes, and though you do not intend to ascend Pilatus from there it makes a good excursion, for lovely walks abound in all directions.

### The Axenstrasse.

As its name indicates, the Axenstrasse is a road that has been hewn out along the mountain side. It extends along the greater part of the lake, and is of unrivalled beauty in its own particular line. It is a grand surface for cyclists and delightful for pedestrians; by the help of the steamers very moderate walkers can enjoy all its charms. This was my plan, by which all fatigue was avoided. Take the convenient steamer, say, to Gersau, get out and walk on to Brunnen. Take a return steamer from Brunnen to Gersau, and walk on to Weggis, where the steamer can be taken again to cross the arm of the lake which runs up to the Zuger See. Another day, take the steamer straight to Flüelen, which is at the end of the lake, and walk back to Brunnen. This is the finest part of the Axenstrasse, and passes by Tell's Chapel. From Brunnen the steamer may be taken home. It is worth taking the train at Flüelen and visiting Altdorf, the scene of Tell's exploit. It is only one and a-half miles from Flüelen, and is a quaint little place, with something of an Italian flavour about its primitive streets.

### Excursions on the South Bank.

Beckenried is a good point to start from, walk over to Seelisburg, about six miles; or, what is easier, take the steamer direct to Treib and walk to Seelisberg, about one mile, and then on to Banin, and from thence home by water. About Buochs, too, the country is charming; there is a pretty walk inland to Stans, or a diligence, which goes several times a day, will take you there in less than an hour; then walk across to Stansstad, only about two miles, and steamer home. Another fascinating excursion is to take boat to Stansstad, and then walk to Sarnen, returning by the Brunnen railway. This is one of the most beautiful excursions of the neighbourhood, but it is rather a long walk. Baedeker, who is seldom at fault, says it takes three hours, and no doubt he is right; but I did it by instalments, taking nearly the whole day, and wandering up and down little sidepaths, and exploring all kinds of fascinating nooks, and it seemed really a very easy walk, but it must be undertaken with circumspection, and not by poor walkers.

### The Zuger See.

One day must be devoted to visiting this lake. The steamer goes all round it, and the little town is very interesting; so is Immensee, which is most beautifully situated at the foot of the Rigi. Arth Goldau, like all these lake villages, is very pretty, but its chief interest is a melancholy one. The railway station now stands where a terrible landslip from the heights of Grippen in 1806 destroyed several hamlets and some hundreds of their inhabitants. By train you can reach the pretty little lake of Lowerz, with its island and ruined castle. There is very much more that I could point out to you around Lucerne, but I must leave it for a future occasion. For those staying longer there are several longer excursions, and some mild climbing quite within the powers of ordinary walkers, but for a short time I am sure it is unadvisable to attempt anything so ambitious. If money will not run to a residence in Lucerne itself I can tell you of several little places round where the terms are very moderate and suited to the humblest purses.

### Minor Appointments.

North Devon Infirmary, Barnstaple.—Miss Duckworth has been appointed Charge Nurse. She was trained at the Preston and Lancashire Royal Infirmary for three years, and has since been charge nurse at Leith Hospital for three years.

Portsmouth Workhouse Infirmary.—Miss Edith Susanah Crisp has been appointed Charge Nurse. She was trained at St. Olave's Union, and has since been nurse at St. Giles's Workhouse Infirmary, and the Fountain Fever Hospital, Tooting.

Bootle Infectious Diseases Hospital, Linacre Lane.—Miss Sophia Colthurst has been appointed Sister. She was trained at Liverpool Royal Infirmary, and has since been night superintendent at Hull Royal Infirmary, and matron at Retford Cottage Hospital.

West Ham Hospital.—Miss Florence Granville has been appointed Night Sister. She was trained at West Ham Hospital, and has since been sister for two years in the women's ward.

